# Meditation-Relaxation (MR Therapy) for Sleep Paralysis: A Pilot Study in Patients With Narcolepsy

**DOI:** 10.3389/fneur.2020.00922

**Published:** 2020-08-12

**Authors:** Baland Jalal, Ludovico Moruzzi, Andrea Zangrandi, Marco Filardi, Christian Franceschini, Fabio Pizza, Giuseppe Plazzi

**Affiliations:** ^1^Behavioural and Clinical Neuroscience Institute and Department of Psychiatry, University of Cambridge School of Clinical Medicine, Cambridge, United Kingdom; ^2^Department of Biomedical and Neuromotor Sciences (DIBINEM), University of Bologna, Bologna, Italy; ^3^Clinical Neuropsychology, Cognitive Disorders and Dyslexia Unit, Department of Neuro-Motor Diseases, Azienda Unità Sanitaria Locale - IRCCS, Reggio Emilia, Italy; ^4^Department of Medicine and Surgery, University of Parma, Parma, Italy; ^5^IRCCS Istituto delle Scienze Neurologiche di Bologna, Bologna, Italy

**Keywords:** sleep paralysis, treatment, meditation-relaxation therapy, narcolepsy, proof-of-concept study

## Abstract

Sleep paralysis (SP) is a condition where a person is paralyzed upon waking or falling asleep. SP afflicts ~20% of people, and is also one of the typical symptoms in narcolepsy. During SP the sleeper may experience hallucinations. Unsurprisingly, SP is associated with great fear globally. To date, there are no published clinical trials or outcome data for treating this condition. However, few non-pharmacological interventions have been proposed, including cognitive behavioral approaches, and case studies showing clinical amelioration with auto-hypnosis and Meditation-Relaxation (MR) therapy. The latter for instance showed positive preliminary results; when applied for 8 weeks it reduced SP frequency and anxiety/worry symptoms. With this paper we aimed to evaluate, with a small-scale pilot study, the efficacy of MR therapy for SP in patients with narcolepsy. Ten patients with narcolepsy and SP were enrolled in the study. Notably, MR therapy (*n* = 6), applied for 8 weeks, resulted in a dramatic decrease in the number of days SP occurred (50% reduction); and the total number of SP episodes (54% reduction) in the last month of the study (demonstrated by large within-group effect sizes); unlike the control intervention (deep breathing) (*n* = 4). These findings are preliminary and exploratory given the small sample. Nonetheless, they represent the first proof of concept at providing empirically-guided insights into the possible efficacy of a novel treatment for frequently occurring SP. Although the study was conducted in patients with narcolepsy we cautiously suggest that the findings may generalize to individuals with isolated SP.

## Introduction

Sleep paralysis (SP) is a state of atonia—skeletal muscles paralysis—occurring at sleep onset and offset. While temporarily immobilized, the person is acutely aware of his/her surroundings (i.e., semi or fully conscious) (1–3). SP commonly occurs in narcolepsy; a sleep disorder involving excessive daytime sleepiness, cataplexy and other untimely manifestations of rapid-eye-movement (REM) sleep such hypnagogic hallucinations and SP (4). SP is also prevalent in the general population, afflicting ~20% of people (1, 5, 6).

Hypnogogic or hypnopompic hallucinations may occur during SP and can involve all sensory modalities (7). Strikingly, SP experiencers often report being terrorized by dangerous bedroom intruders (8, 9). Supernatural interpretations of SP are found worldwide and often reflect the cultural background of the population in question.

In terms of neurophysiology, SP represents a REM sleep parasomnia reflecting the dissociation between REM sleep and wakefulness, with the concurrent presence of REM sleep muscle atonia, wakefulness consciousness, and, eventually REM dream mentation (1). SP occurs at the transition between wakefulness and REM sleep in narcolepsy patients—or in otherwise healthy subjects (10). Polysomnographic studies disclosed the co-occurrence of wakefulness features and REM sleep during SP episodes (11, 12). SP ranges in duration from a few seconds up to 20 min, although under paralysis patients are not accurate in such subjective judging of the passing of time (13).

SP in the general population may be triggered by sleep deprivation (14), and is more frequent in psychiatric conditions like post-traumatic stress disorder (15). Unsurprisingly, SP is linked to excessive fear, which persists after continual exposure to the event (16, 17).

Notwithstanding the trepidation and anxiety associated with the experience, to date there are no published clinical trials or outcome data for treating this condition. However, few non-pharmacological interventions have been proposed including cognitive behavioral approaches (18, 19). Moreover, case studies have shown clinical amelioration with auto-hypnosis (13), and Meditation-Relaxation (MR) therapy (20). Only a limited number of patients report a severe enough condition that would allow for the off-label use of antidepressants (21, 22). Thus, most sufferers would benefit from a non-pharmacological intervention.

MR therapy—a simple (non-invasive) and *direct* 4-step psychological intervention—was recently proposed for SP (20). The aim of this pilot study was to explore the preliminary efficacy of MR therapy for SP (applied for 8 weeks) in patients with narcolepsy, compared to deep breathing techniques. A secondary aim was to examine features of SP in narcolepsy and its link to psychopathology (mood/anxiety).

## Methods

### Participants

The study was conducted at the Center for Narcolepsy of the University of Bologna/IRCCS Istituto delle Scienze Neurologiche di Bologna, Italy and approved by the ethics committee at the CE-AVEC Independent Ethical Committee, Area Vasta Emilia Centro. Patients provided written consent. Participants met criteria for narcolepsy (type 1 and 2) based on polysomnography, Multiple Sleep Latency Test (MLST) and CSF hypocretin level testing. Patients had to have experienced SP at least four times in the last month. The study included ten patients (female = 40%; age: *M* = 27.8, *SD* = 12.2) (Two patients were <18 years old; one turned 18 during the study [in both cases written parental approval was obtained]).

### Measures

#### SP Assessment

SP was assessed using the Sleep Paralysis Experiences and Phenomenology Questionnaire (SP-EPQ) (23–25).

#### Daily Journal (Outcome Variables)

Each day during the study (12 weeks), participants rated their SP occurrence, perceived duration (seconds), fear associated with SP, and disturbance caused by hallucinations (on a ten-point scale).

#### Baseline Measures of Mood/Anxiety

Participants completed the following validated measures: the Beck Depression Inventory-II (BDI-II) (26), Spielberger State-Trait Anxiety Inventory (STAI-S/T) (27), and Penn State Worry Questionnaire (PSWQ) (28). The Italian versions were completed (29–31).

#### Intervention

*MR therapy* is a psychological treatment for SP, comprised of the following steps applied *directly* during the attack: Step I: *Reappraisal of the meaning of the attack*; Step II: *psychological and emotional distancing*; Step III: *inward focused-attention meditation*; Step IV: *Muscle relaxation* [for details see (20)]. The control intervention was identical, except participants engaged in deep breathing instead; entailing slow deep breaths, while repeatedly counting from 1 to 10. This is an active control (breathing-distraction exercise) rather than a placebo.

### Procedure

SP experiencers were recruited at the Outpatient Clinic for Narcolepsy and assigned to either the MR therapy or control group (open label study design). Participants were orally administered the SP-EPQ to assess their SP (e.g., frequency and features), and were instructed to keep daily a journal for 4 weeks as a baseline assessment of SP occurrence, duration and emotions (see above) and email this to the researcher each week. After the 4 weeks, participants completed mood/anxiety questionnaires and were taught the therapy techniques (i.e., practiced them with the experimenter), and instructed to rehearse these during ordinary wakefulness (simulating a SP attack), twice a week for 15 min. These instructions (e.g., treatment techniques) were administered in person or via video call. Participants then initiated the treatment which lasted 8 weeks. They kept the daily journal during the entire study period (12 weeks).

### Statistics

Exploration of the link between SP frequency and hallucinatory-induced disturbance and mood/anxiety symptoms were analyzed using a *Pearson* correlation test. Independent sample *t-tests* were used to analyze baseline group differences on outcome variables. Specific hypotheses pertaining to MR therapy and control intervention were analyzed using focused directional paired sample *t-*tests.

For test of a priori hypotheses (i.e., one-tailed *t-*tests), the Benjamini and Hochberg's (32) false discovery rate (FDR) was applied to correct for potential Type I errors. The FDR was set at *q* <0.15, based on guidelines in the field (33). The corrected significance level/critical value was 0.045. Displayed *P*-values are raw; designated significant/non-significant *after* applying the correction (34). Exploratory/baseline analyses were not corrected for multiple comparisons, which is unnecessary (35).

The distribution of residuals was checked using the Shapiro-Wilk test and Q-Q plots for all measures. Residuals sometimes departed from normality. Given the *F*/*t*-test is overall robust to violations (36), the data were analyzed with those caveats.

## Results

### Baseline/Pre-treatment Period (Initial 4 Weeks)

#### Features of SP

The patients (*n* = 10) experienced SP on average 8.30 days (*SD* = 4.57) total, and 10.20 (*SD* = 7.38) SP episodes in the last month (i.e., during the baseline period). They reported being paralyzed on average 289.89 sec (*SD* = 448.01). Patients tended to hallucinate during SP (on average in 66% [SP with hallucinations/total SP] of cases in the last month). SP hallucinations often occurred upon awakening from sleep (51%), and less frequently upon falling asleep (14%); a proportion of SP episodes occurred during “sleep” (i.e., neither at onset nor offset; 24%).

#### SP, Hallucinations and Mood

The number of SP episodes in the last month did not correlate with depression, anxiety and worry symptoms (i.e., during the baseline period). Disturbance caused by SP hallucinations correlated with depression (*r* = 0.69, *p* = 0.039) and worry symptoms (*r* = 0.69, *p* = 0.039) unlike anxiety (there was a trend for trait anxiety: *r* = 0.58, *p* = 0.099) (one patient did not complete these questionnaires: *n* = 9).

### Treatment

#### Baseline Difference

Participants did not differ on outcome variables (fear, perceived duration of SP, and disturbance caused by hallucinations) except that participants in the MR therapy group reported a greater number of days with SP (*t*_5.24_ = 4.14, *p* = 0.008) and total number of SP (*t*_5.09_ = 3.14, *p* = 0.025) in the last month (during the baseline period).

#### MR Therapy Pre-post

There was a reduction in the number of days with SP (*t*_5_ = 4.68, *p* = 0.0025, one-tailed, *d* = 1.91) and total number of SP (*t*_5_ = 3.86, *p* = 0.006, one-tailed, *d* = 1.57) in the last month pre-post MR therapy. The average fear of SP (*t*_5_ = 0.57, *p* = 0.298, one-tailed, *d* = 0.23) and perceived duration of SP (*t*_5_ = 1.33, *p* = 0.121, one-tailed, *d* = 0.54) in the last month were unchanged; there was a notable tendency toward reductions in the disturbance caused by hallucinations (*t*_5_ = 2.01, *p* = 0.051, one-tailed, *d* = 0.82).

**Figure 1:**
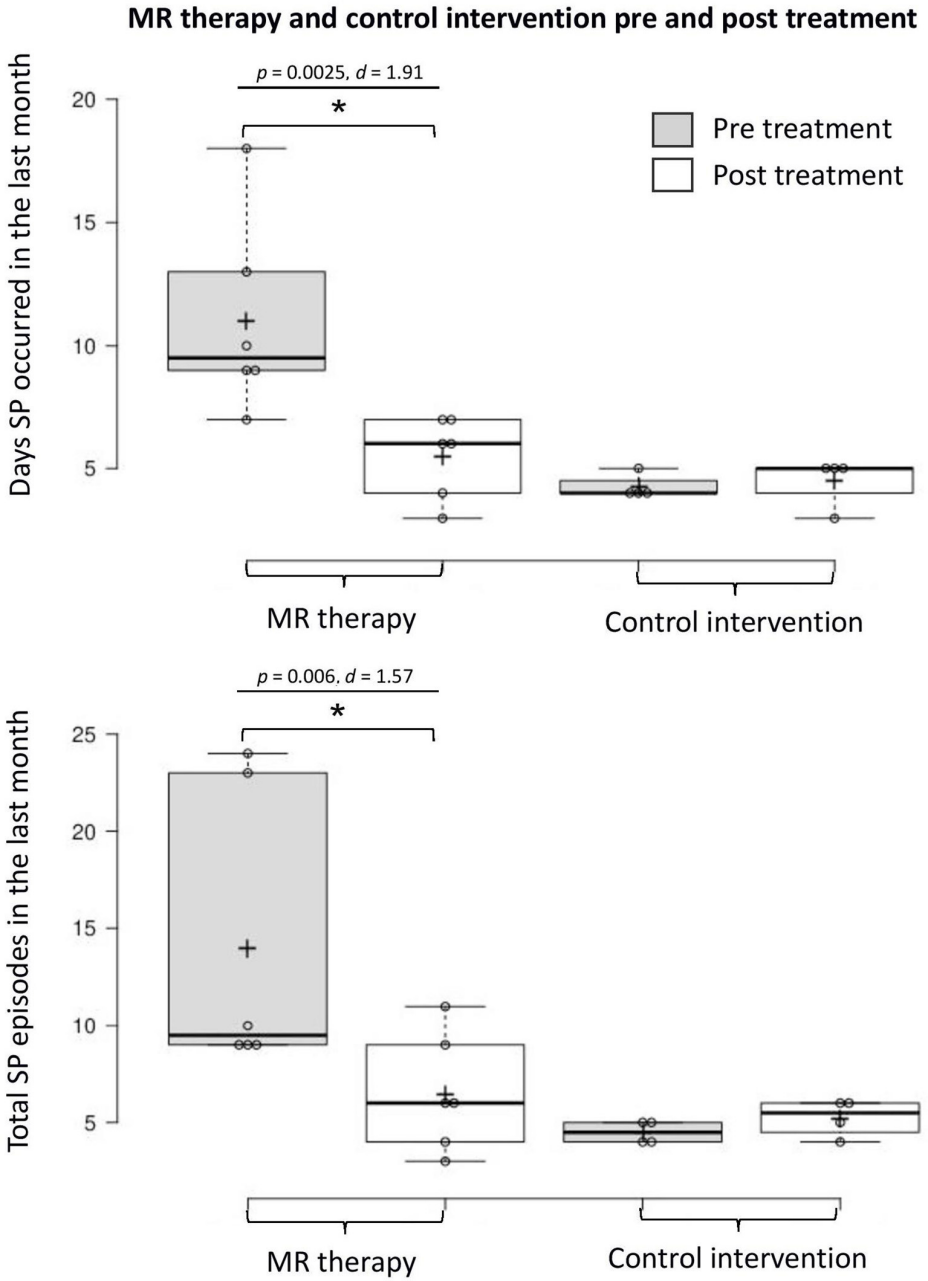
Days with SP and total number of SP in the last month pre-post MR therapy and control intervention (deep breathing). “Pre treatment” reflects the baseline assessment period (4 weeks before commencing treatment); “Post treatment” reflects the last 4 weeks of treatment (week 5 to 8 of the treatment period). *Denotes a statistically significant result.

**Figure 2:**
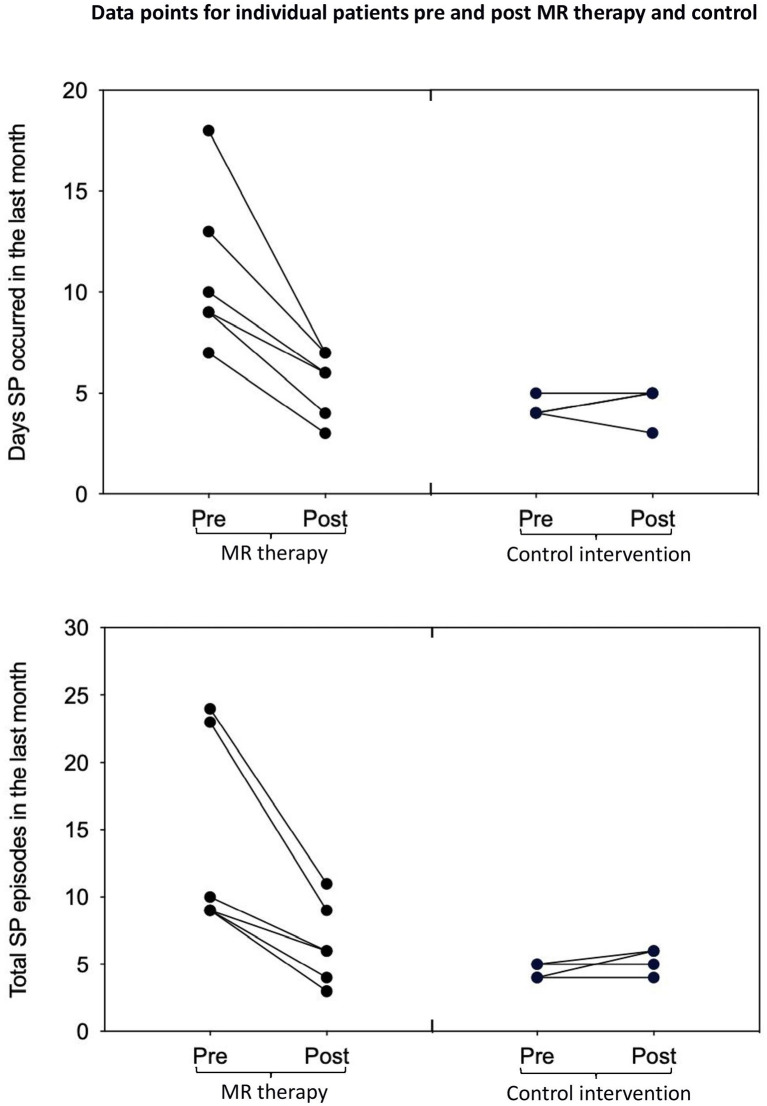
Days with SP and total number of SP in the last month pre-post MR therapy and control intervention (deep breathing). “Pre treatment” reflects the baseline assessment period (4 weeks before commencing treatment); “Post treatment” reflects the last 4 weeks of treatment (week 5 to 8 of the treatment period).

In the control condition, the number of days with SP (*t*_3_ = −0.52, *p* = 1.28, one-tailed, *d* = −0.26) and total number of SP (*t*_3_ = −1.57, *p* = 0.44, one-tailed, *d* = −0.78) in the last month; fear (*t*_3_ = 2.80, *p* = 0.136, one-tailed, *d* = 1.40) and perceived duration (*t*_3_ = −0.83, *p* = 0.936, one-tailed, *d* = −0.41) of SP, and disturbance caused by hallucinations in the last month (*t*_3_ = −0.92, one-tailed, *p* = 0.850, *d* = −0.46) were unchanged [Means and standard deviations are displayed in Table 1; key findings are shown in Figure 1; data points plotted for individual patients [pre-post treatment] are displayed in Figure 2].

**Table 1:** Means and standard deviations for outcome variables pre and post intervention.

**Condition**	**MR therapy (*****n*** **=** **6)**				**Control intervention (*****n*** **=** **4)**			
	**Pre**		**Post**		**Pre**		**Post**	
	***M***	**(*SD*)**	***M***	***(SD*)**	***M***	**(*SD*)**	***M***	**(*SD*)**
Days with SP	11.00	(3.95)	5.50	(1.64)	4.25	0.50	4.50	1.00
Total SP	14.00	(7.38)	6.50	(3.02)	4.50	0.58	5.25	0.96
Fear of SP	4.22	(2.41)	3.63	(1.47)	2.15	2.67	1.67	2.61
Duration of SP (seconds)	361.61	564.49	200.66	273.30	182.31	212.94	303.40	498.83
Hallucination disturbance	7.29	3.02	4.79	2.04	3.96	3.27	4.48	4.00

*Note: M, mean; SD, standard deviation; n, sample size. “Pre” = average daily journal ratings during the baseline assessment period (4 weeks before commencing treatment); “Post” = ratings during the last 4 weeks of treatment (i.e., week 5 to 8 of the treatment period). “Days with SP” = days that SP occurred; “total SP” = total number of SP occurrences. “Fear of SP” = fear associated with SP; “Hallucination disturbance” = disturbance caused by SP hallucinations. “Fear of SP” and “hallucination disturbance” were rated on a ten-point Likert scale*.

## Discussion

This small-scale pilot study yields novel findings with clinical implications. MR therapy resulted in a dramatic decrease in the number of days SP was experienced (50% reduction), and total number of episodes (54% reduction) in the last month of the study (illustrated by large within-group effect sizes). There was also a noteworthy (*p* = 0.05) trend toward reductions in the disturbance caused by SP hallucinations (a 34% reduction in self-report ratings, with a large effect size). Given the small sample size, *p*-values should be interpreted cautiously, and effect sizes should take priority. These effects were not observed in the control intervention where the occurrence of SP and associated negative emotional states were unchanged. Overall, these preliminary findings suggest that MR therapy potentially constitutes a promising treatment option for SP; pending confirmation in randomized clinical trials.

That MR therapy was applied by the patients in their home environment is notable. Given the simplicity of MR therapy, it lends itself to internet- and smartphone-based delivery; e.g., where therapy instructions are administered online, and remotely overseen by a clinician, who can intervene promptly when necessary. As noted, in this study, in some cases for logistic reasons therapy was delivered via video call. In brief, this low-cost and accessible intervention may facilitate early intervention (prevent SP from becoming chronic and possibly spiral into psychopathology), and is potentially suitable for poorly resourced settings and low-income countries with restricted access to health care. This approach may be of valuable utility also in periods such as the current COVID-19 pandemic when face-to-face interventions are limited.

In the current study, consistent with research in general populations, disturbance caused by SP was associated with elevated psychopathology (depressive and worry) symptomatology. These findings are of clinical value. They suggest that SP-associated psychological disturbance may contribute to overall emotional instability in narcolepsy (37). It is also conceivable that elevated psychopathology symptoms might increase the tendency to catastrophize SP hallucinations. As such, applying psychological treatments to specifically target catastrophic cognitions associated with SP-related fears (e.g., combined with *direct* SP treatments) may help improve these patients' emotional well-being (reduce distress) and effectively their global functioning (38).

This novel venture sheds light on the experience of SP in narcolepsy. Reminiscent of the general population, patients' self-estimated duration of SP was ~5 min, occurred most often at sleep offset, and a comparable proportion of individuals hallucinated during the event [e.g., 66 vs. 72% in the general Italian population, see (25)]. Taken together, SP in narcolepsy and the general population appear to be phenomenologically indistinguishable, and further studies are needed to directly compare their features.

The patients in this study relied on a daily journal to rate their SP occurrence, duration and emotions which constitutes a strength; i.e., that they kept a journal not only during the treatment (8 weeks), but also prior during baseline assessment (4 and 12 weeks total). In contrast, retrospective accounts of such physiological and psychological states may lack precision. The study has limitations. Since it was conducted in narcolepsy patients the findings may possibly generalize to isolated SP patients; this should be explored further (given that SP in narcolepsy, unlike ISP, is within the context of hypocretin deficiency typical of narcolepsy). Also, while the reduction in SP following MR therapy was striking (as noted, a 54% monthly reduction), we cannot rule out that pre-existing higher frequency/severity of SP may have accounted for its superiority relative to the control intervention. Indeed, future research (randomized controlled trials) should actively match groups based on clinical severity to rule out a threshold effect; that we did not do so constitutes a limitation. In this study, patients were assigned to the two groups in a non-randomized, open label fashion; however, future studies should ensure proper allocation concealment to prevent potential selection bias. Finally, future sufficiently powered studies should assess the effect of therapy on a greater number of outcome variables (e.g., related to hallucinations and mood). We hasten to add that these findings are preliminary given the small sample. Nonetheless, they represent the first attempt at providing (empirically-guided) insights into the possible efficacy of a novel treatment for chronic SP—a condition all too often overlooked by clinicians and researchers alike.

## Data Availability Statement

The raw data supporting the conclusions of this article will be made available by the authors, without undue reservation.

## Ethics Statement

The studies involving human participants were reviewed and approved by the CE-AVEC Independent Ethical Committee, Area Vasta Emilia Centro. Written informed consent to participate in this study was provided by the participants and/or the participants' legal guardian/next of kin.

## Author Contributions

BJ designed the study, performed the data analysis, and wrote the manuscript. AZ designed the study, analyzed the data, and edited the manuscript. LM recruited and tested patients (e.g., administered the therapy), prepared the data for analysis, and edited the manuscript. FP recruited patients and edited the manuscript. GP designed the study, recruited patients, and edited the manuscript. MF recruited patients, and reviewed the manuscript. CF recruited patients and reviewed the manuscript. All authors read and approved the submitted version.

## Conflict of Interest

BJ will receive royalties from Cambridge University Press commencing 2020. GP has served as consultant for UCB, Bioprojet, Jazz, Idorsia. The remaining authors declare that the research was conducted in the absence of any commercial or financial relationships that could be construed as a potential conflict of interest.
